# Electronic hand hygiene monitoring systems: Not worth the costs

**DOI:** 10.1017/ash.2022.264

**Published:** 2022-07-25

**Authors:** Michelle E. Doll, Jacob W. Pierce

**Affiliations:** Virginia Commonwealth University Medical Center, Richmond, Virginia

## Abstract

Electronic hand hygiene monitoring systems allow the collection of large volumes of data. However, significant resources are required to validate and maintain these systems. Additionally, data are lacking on the correlation with clinically important outcomes. Direct observation of hand hygiene remains the gold standard for monitoring hand hygiene compliance.

Electronic hand hygiene monitoring systems (EHHMSs) offer a high-tech solution to suboptimal healthcare worker hand hygiene performance. In an era of increasingly stretched resources for infection prevention and control (IPC) programs, EHHMSs promise an automatized approach to hand hygiene programs, more robust data collection, and improved compliance. However, potential purchasers of these systems should not underestimate the level of investment of resources required to maintain functionality and thus contribute to improved hand hygiene (HH) performance.

## The promise of EHHMSs

EHHMSs have several advantages over traditional observation or “secret shopper” HH monitoring programs. The most striking is the volume of data that these systems capture. Facilities can expand from a few thousand HH observations per month to 200,000–300,000 observations per month.^1^ Data are collected on nights, weekends, and holidays when observers typically do not capture as much data. In addition, many of these systems can provide real-time feedback to providers in the event of a missed opportunity. EHHMS data may be less subjective than direct observations and are thought to be less susceptible to the Hawthorne effect.^2^ EHHMSs also claim to increase HH compliance and ultimately decrease HAIs. However, evidence to support these outcomes is limited and may depend on the group of providers using these technologies, the specific technology type, or details of the implementation. Yet there is enthusiasm for these systems, based on these promises. For example, the Leapfrog Group has focused heavily on EHHMSs in their recent surveys, recommending that institutions strongly consider implementing these systems.^3^ However, the assumption that every hospital can successfully implement one of these systems may overestimate available resources as well as the ability of these systems to change healthcare provider HH practices.

## System-defined compliance

When considering potential benefits of EHHMS implementation, it is important to consider the nature of the data they provide. EHHMS compliance is defined and influenced by the system itself. This system-defined compliance (SDC)^4^ can be adjusted based on inputs provided to the system. Providers can be given more or less time to perform HH, the area in which HH is expected (ie, at room entry and/or exit, or a patient zone) can be expanded or contracted, or the time the provider is in the patient zone before HH is expected can be increased or decreased. Permissive settings will ensure higher system-defined compliance, whereas restrictive settings will lower SDC.

In this setting, observer data remain critically important to link SDC from EHHMS to actual clinical events. Yet SDC is often the only outcome reported in published studies. Is reported SDC truly reflecting provider HH behavior?

This disconnect between SDC and actual observed provider HH behavior was highlighted in a study by Pineles et al^5^ in which an EHHMS was implemented in 2 phases. In phase 1, a simulated test of the technology was conducted in which researchers walked the units triggering the technology on a planned path. The expected number of HH encounters on that path was compared to the data recorded in the EHHMS. In phase 2, the researchers observed providers in their clinical workflows The data from the EHHMS were compared to direct observation data. The technology performed well in the planned path validation exercises but poorly in capturing HH as part of a clinical workflow. Specifically, the technology had issues detecting providers in the wrong room, wrongly attributing HH events to another provider, or capturing badges when the provider was turned.^5^

## Why validate?

Limper et al^6^ suggest that validation of EHHMS performance should be a routine part of implementation, and they note that it is rarely described in studies evaluating the effects of these systems. These researchers also suggested that the vendors should be invested in doing these types of validations of their equipment. In reality, planned path validations will most likely be the responsibility of an IPC team, and it is necessary to perform path validations on a routine basis once one of these technologies is in place. When pieces of the system stop working because of dead batteries or other malfunctions, providers notice and complain. It is then up to the internal program team to evaluate and fix the issues. Ideally, to maintain provider trust in the technology, the IPC team can identify issues before complaints occur.

In our experience, a HH program manager tests >2,000 dispensers through planned path validations and performs 100 instances of behavioral validation over a 2-year period. Thus, ongoing validation efforts require a significant investment of time and resources.

In addition, not all systems demonstrate a high level of accuracy. Our group has previously published a study on the accuracy of 2 systems pilot-tested at our institution.^7^ We reported significant disparity in accuracy between the 2 systems, highlighting the importance of validation and ensuring a given system is a good match for the institution prior to implementation.

## Financial costs

EHHMSs are associated with significant costs. The up-front purchasing costs can range from $500 per bed to >$2,000 per bed. Many companies will have an ongoing subscription fee for maintenance of the system, including replacements of badges, etc. Up-front purchasing costs are just one aspect of the system that will need to be budgeted. In a large facility, a dedicated full-time equivalent (FTE) to the HH program will be needed to answer concerns and questions, distribute badges, identify, and fix mechanical issues including replacement of dead batteries. Some of these tasks could be delegated to non-IPC team members; however, dedicated personnel committed to the HH mission are required to ensure that maintenance, validation, and data analysis and interpretation are completed.

## Human resources

Much of the literature supporting the potential of an EHHMS to increase HH is based on limited implementation at a single center where participants are highly motivated. The gold standard for evaluating the effectiveness of an EHHMS to prevent HAIs would require multicenter randomized controlled trials. To reflect real-world practices, such studies should engage the entire institution, including those who may be less motivated. A comprehensive assessment would need to allow sufficient follow-up to account for declines in compliance after initial implementation and to account for maintenance requirements of the system. The only successful accounts of implementation leading to improvement in HH across an organization paired the technology with extensive educational campaigns and other IPC resources, raising the question of what portion of this intervention actually changed provider behaviors.^8^ It should not be assumed that the same results would follow from simply putting an EHHMS in place at a facility.

## Opportunity costs

An EHHMS forces discussions about HH compliance as well as data review in the form of evaluation and feedback. These are key components of a multimodal HH program. However, physician champions for these systems invest significant time and resources explaining data from the EHHMS and troubleshooting individual units, along with the IPC team.

More than 10 years ago, Wright et al^9^ described the discrepancy between the increasing task load within IPC and available workforce.^9^ The coronavirus disease 2019 (COVID-19) pandemic has further stretched already limited resources within IPC programs. Implementation of an EHHMS likely requires sacrificing resources from core IPC activities, and the impact on key priorities of the program must be considered. Direct observation of HH must not be sacrificed to implement an EHHMS because observations provide the most accurate understanding of provider behaviors and HH performance in real-world clinical encounters.

Presently, EHHMSs cannot be universally recommended for all health systems. The key potential advantage of individual accountability is limited by concerns regarding accuracy and clinical context of the data. Furthermore, data to support a linkage between EHHMS implementation and important clinical outcomes of HAI prevention remain limited.

Observation data remain the gold standard for tying individual HH events to diverse patient-care activities, and these data are necessary in the interpretation of SDC data from an EHHMS, even when present. Given limited resources within IPC programs, facilities should carefully consider the opportunity costs associated with EHHMS implementation. If institutions, choose to adopt these systems, they must consider how the data will be utilized as a part of a multifaceted HH program.
